# Sleep postures monitoring based on capacitively coupled electrodes and deep recurrent neural networks

**DOI:** 10.1186/s12938-022-01031-5

**Published:** 2022-10-13

**Authors:** Shun Peng, Yang Li, Rui Cui, Ke Xu, Yonglin Wu, Ming Huang, Chenyun Dai, Toshiyo Tamur, Subhas Mukhopadhyay, Chen Chen, Wei Chen

**Affiliations:** 1 Center for Intelligent Medical Electronics, School of Information Science and Technology, Fudan University, Shanghai, 200433 China; 2 Department of Infectious Disease, Imperial College London, London, UK; 3 Computational Systems Biology, Division of Information Science, Nara Institute of Science and Technology, Nara, Japan; 4 Institute for Healthcare Robotics, Waseda University, Tokyo, 162-0041 Japan; 5 School of Engineering, Macquarie University, Sydney, NSW 2109 Australia; 6 Human Phenome Institute, Fudan University, Shanghai, 201203 China

**Keywords:** Capacitively coupled electrode, Sleep posture, Capacitive electrocardiogram, Recurrent neural network

## Abstract

**Background:**

Capacitively coupled electrode (CC electrode), as a non-contact and unobtrusive technology for measuring physiological signals, has been widely applied in sleep monitoring scenarios. The most common implementation is capacitive electrocardiogram (cECG) that could provide useful clinical information for assessing cardiac function and detecting cardiovascular diseases. In the current study, we sought to explore another potential application of cECG in sleep monitoring, i.e., sleep postures recognition.

**Methods:**

Two sets of experiments, the short-term experiment, and the overnight experiment, were conducted. The cECG signals were measured by a smart mattress based on flexible CC electrodes and sleep postures were recorded simultaneously. Then, a classifier model based on a deep recurrent neural network (RNN) was proposed to distinguish sleep postures (supine, left lateral and right lateral). To verify the reliability of the proposed model, leave-one-subject-out cross-validation was introduced.

**Results:**

In the short-term experiment, the overall accuracy of 96.2% was achieved based on 30-s segment, while the overall accuracy was 88.8% using one heart beat segment. For the unconstrained overnight experiment, the accuracy of 91.0% was achieved based on 30-s segment, while the accuracy was 81.4% using one heart beat segment.

**Conclusions:**

The results suggest that cECG could render valuable information about sleep postures detection and potentially be helpful for sleep disorder diagnosis.

## Introduction

Capacitively coupled electrode (CC electrode) has been widely studied in the last decade because of its user-friendly characteristic [1]. It senses the electrical potential on the body surface through coupling capacitance between the skin and electrodes, which allows measuring physiological signals over the clothes without subject awareness. Since the skin–electrode impedance is very large in this non-contact setting, a voltage buffer is usually designed at the signal input end of the hardware to increase its input impedance and enhance the anti-interference ability and load capacity of signal acquisition [2]. Compared with the conventional wet electrode, CC electrode has many advantages. For example, it works without the need of skin preparation and the risk of skin irritation [3], and can be used repeatedly [4]. Therefore, CC electrode has been embedded into diverse objects including toilet seats [5], bathtubs [6], driver’s seats [7], chairs [2], bedsheets, and mattresses [8]. It has found a wide range of applications in many scenarios, such as sleep [9], car driving [10] and exercise monitoring [11], as well as during office work and other daily life [12]. Capacitive electrocardiogram (cECG) measurement is the most common implementation of CC electrodes, from which heart-related parameters including RR interval, heart rate (HR), and heart rate variability (HRV) are often calculated [13]. Based on cECG and heart-related parameters, further researches are carried out, including monitoring of arrhythmias such as premature ventricular contraction [14], diagnosis of cardiovascular disease (e.g., acute myocardial infarction [15]), fatigue detection [16], and man–machine emotional communication [17]. It is worth noting that the lead modes of cECG measurement in sleep monitoring are not standard leads, since CC electrodes are usually fixed in the mattress. It senses ECG signal from different positions of the body surface when the subjects lie on the bed in different sleep postures. Therefore, cECG signals obtained from mattress may contain information that reflects the current sleep posture of the subjects and could be used for sleep posture recognition.

Sleep posture recognition, as an effective method for sleep quality assessment, has been extensively studied. It has been proved to be helpful in pressure ulcer prevention [18] as well as diseases diagnoses and treatment [19, 20]. The most intuitive and visual way to recognize sleep posture is camera-based approaches [21], including motion capture, depth scans, and infrared imaging. However, the main problem with camera-based approaches is the invasion of user privacy. Another common way of sleep posture recognition is based on wearable devices [22], such as accelerometers, magnetometers, and gyroscopes. These sensors can monitor sleep posture accurately without privacy concerns and are even used in polysomnography (PSG) which is the golden standard of sleep monitoring. However, all of them need to be attached to the body, leading to discomfort and inconvenience during sleep. Most recently, sleep posture recognition using a pressure sensor array on the bed, also known as the pressure map [23], has aroused extensive attention. It identifies the sleep postures by the pressure distribution map of the subject lying on bed and the state-of-the-art accuracy has already exceeded 97% under the high-density sensor array [24, 25]. However, a large number of sensors lead to a huge increase in the complexity of the system and the burden of signal processing.

The only study we found on sleep posture recognition based on cECG was proposed by Lee et al. [26]. Capacitive ECG signals from 13 subjects were collected using 12 CC electrodes and a conductive textile sheet. Then, based on the morphological characteristics of the QRS complex and three machine learning algorithms, the highest accuracy of the sleep posture recognition was 98.4%. However, the CC electrodes they used were made of a printed circuit board, which may cause discomfort and seriously disturb natural sleep. In addition, too many electrodes may lead to a jumble of wires on the bed. Finally, the channel selection caused by small size of CC electrode increases the complexity and difficulty of signal preprocessing. Therefore, the performance of sleep monitoring may decrease if the channel selection is performed on-line in real-time monitoring.

In this study, a smart mattress embedded with only three flexible electrodes was presented. And a classifier model based on deep recurrent neural network (RNN) was proposed to distinguish sleep postures. We designed two sets of experiments, the short-term experiment and the overnight experiment to evaluate the performance of the system. To the best of our knowledge, it is currently the first work that recognizes sleep posture using ECG signal measured by flexible CC electrode. The main contributions of this work can be summarized as follows:

- 1) A smart mattress based on flexible CC electrode was designed to monitor sleep posture. The system only contains three electrodes embedded in the mattress, reducing the complexity of the sleep posture monitoring system and facilitating its implementation. It can measure physiological signals comfortably, unobtrusively and without privacy concerns.
- 2) The bidirectional Long Short-Term Memory (biLSTM) network, was used to classify sleep posture. The proposed network achieved considerably high accuracy without any manual feature extraction.
- 3) The overnight experiment was carried out to evaluate the performance of the proposed system in a real sleep scenario. Results suggested that the mattress could be potentially promising in long-term sleep monitoring.

## Results

### Short-term experiment results

Leave-one-subject-out cross-validation (LOSOCV) was introduced to verify the reliability of the classifier model. That is, testing set contained only one subject’s data in each validation and the data of each subject was used once for testing. So, the process was repeated 15 times in the whole data set. The classifier performance was calculated by three indices including sensitivity ($\mathrm{SEN}$), accuracy ($\mathrm{ACC}$) and Cohen’s kappa coefficient ($\mathrm{Kappa}$), as defined below were used to evaluate the model performance:

1 $${\text{SEN}} = \frac{{{\text{TP}}}}{{{\text{TP}} + {\text{FN}}}} \times 100\% ,$$

2 $${\text{ACC}} = \frac{{{\text{TP}} + {\text{TN}}}}{{{\text{TP}} + {\text{TN}} + {\text{FP}} + {\text{FN}}}} \times 100\% ,$$

3 $${\text{Kappa}} = \frac{{p_{o} - p_{e} }}{{1 - p_{e} }},$$

wherein $\mathrm{TP}$, $\mathrm{FP}$, $\mathrm{TN}$, $\mathrm{FN}$ represent “true positive”, “false positive”, “true negative” and “false negative”, respectively, while ${p}_{\mathrm{o}}$ and ${p}_{\mathrm{e}}$ stand for the observed and the expected agreement, respectively. The calculation method of kappa value can be found in [27].

**Fig. 1:**
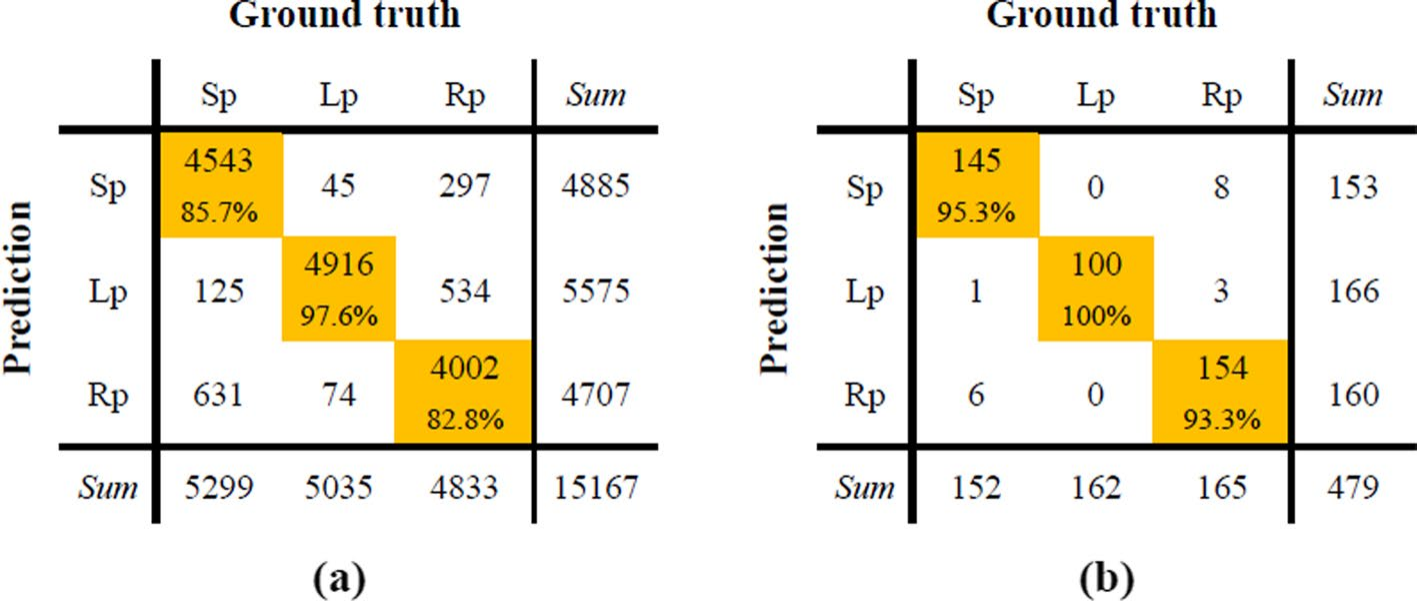
Confusion matrices of sleep posture prediction in short-term data where the length of each data segment is one heat beat (**a**) or 30 s (**b**)

The confusion matrix of the short-term sleep posture prediction is shown in Fig. 1. Table 1 summarizes the posture classification performances of each subject and the whole dataset when the data segment length is one heat beat. Although the sensitivity of several cases (such as right lateral of subject 10 and supine of subject 15) was not very high, a relatively good result was obtained on the average sensitivity, especially in left lateral posture with average sensitivity of 97.6%. Besides, an average accuracy of 88.8% was acquired, proving the overall recognition ability of the proposed method for three sleep postures. The average kappa value of 0.831 showed an almost perfect consistency between ground truth and prediction.

**Table 1:** Performances of sleep posture classification using 1 heart beat segment

Subject number	SEN (%)			ACC (%)	Kappa
	Supine	Left	Right		
1	94.8	100.0	99.4	97.8	0.965
2	97.8	97.5	78.1	91.2	0.867
3	99.7	100.0	95.5	98.4	0.976
4	92.5	98.9	93.6	95.0	0.925
5	96.9	72.6	80.5	83.5	0.752
6	80.3	99.4	71.3	84.3	0.763
7	97.0	100.0	75.8	91.0	0.864
8	81.2	99.1	79.9	86.5	0.796
9	81.5	99.4	79.7	86.6	0.799
10	97.1	100.0	33.2	77.9	0.665
11	98.3	100.0	98.3	98.9	0.983
12	98.4	99.4	66.7	88.2	0.823
13	60.0	100.0	93.5	84.7	0.77
14	57.4	97.6	98.3	84.5	0.767
15	53.1	99.4	98.0	83.4	0.752
Average	85.7	97.6	82.8	88.8	0.831

Table 2 summarizes the posture classification performances using 30-s-length segment. The sensitivities of three sleep postures were 95.3%, 100% and 93.3%, respectively, while the average accuracy and kappa is 96.2% and 0.943.

**Table 2:** Performance indices of sleep posture classification using 30-s segment

Subject number	SEN (%)			ACC (%)	Kappa
	Supine	Left	Right		
1, 2, 3, 4, 5, 6, 7, 8, 9, 11	100.0	100.0	100.0	100.0	1.000
10	100.0	100.0	10.0	70.0	0.550
12	100.0	100.0	90.0	96.7	0.950
13	90.0	100.0	100.0	96.7	0.950
14	70.0	100.0	100.0	90.0	0.850
15	70.0	100.0	100.0	90.0	0.850
Average	95.3	100.0	93.3	96.2	0.943

### Overnight experiment results

**Fig. 2:**
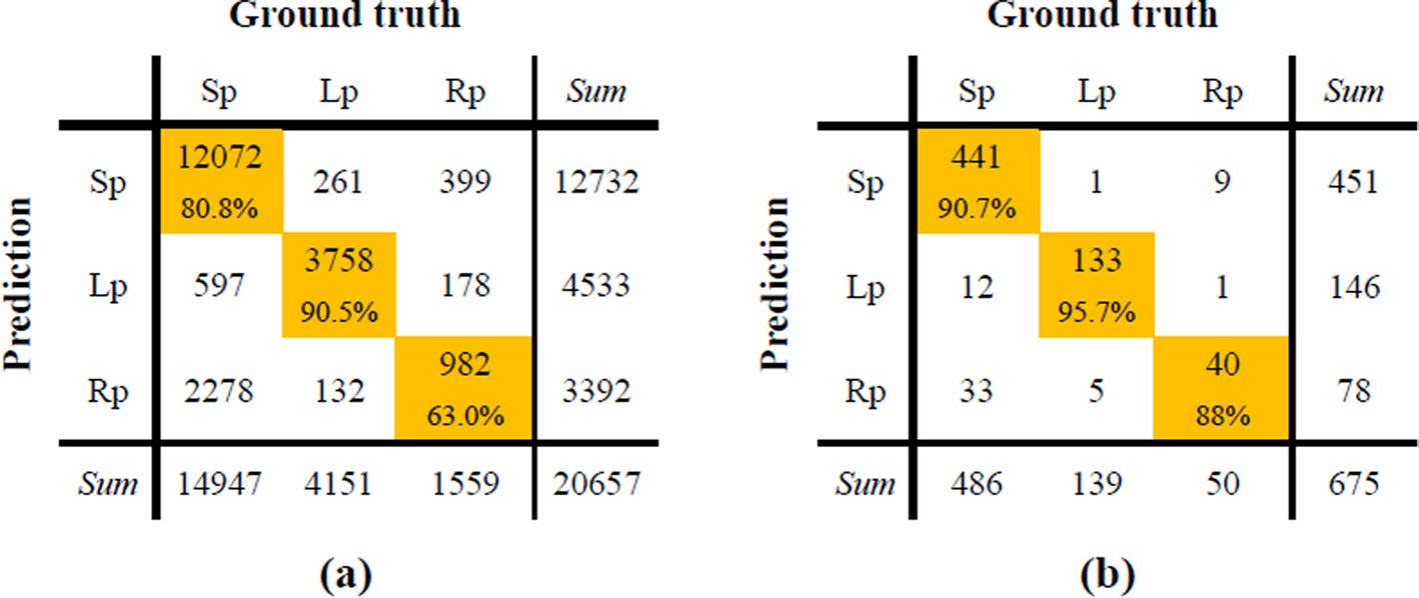
Confusion matrices of sleep posture prediction in overnight data where the length of each data segment is one heat beat (**a**) or 30 s (**b**)

The confusion matrix of the overnight sleep posture prediction is shown in Fig. 2. And the performance indices including sensitivity, accuracy and kappa are shown in Table 3. The sensitivities of three sleep postures using one heart beat segment on were 80.8%, 90.5% and 63.0%, respectively, while the average accuracy and kappa is 81.4% and 0.626. The performance was significantly improved based on 30-s segment, with an accuracy of 91.0% and a kappa of 0.806.

**Table 3:** Performance indices of sleep posture prediction in overnight data

The length of segment	SEN (%)			ACC (%)	Kappa
	Supine	Left	Right		
1 heart beat	80.8	90.5	63.0	81.4	0.626
30 s	90.7	95.7	80.0	91.0	0.806

## Discussion

Sleep quality assessment has received increasing attention in recent years [28–31]. As mentioned in Section I, sleep posture plays an important role in sleep monitoring. Previous studies have proven that it has a certain influence on the incidence of diseases such as bedsores, sleep apnea syndrome, and carpal tunnel syndrome. Recently, unobtrusive monitoring has become an important direction in sleep monitoring research [32], which sets stricter requirements on the flexibility of sensors and the convenience of the system. A large number of sensors in a system may cause hardware complexity and wire clutter. This increases the instability of measurement under unconstrained sleep for more than 6 h, and disturbs users’ natural sleep. In this work, a smart mattress based on flexible conductive fabric which has only three CC electrodes was used for sleep posture recognition. It provided an inexpensive solution to facilitating the convenience of operation, increasing the stability of the measurement, and improving comfort of the subjects.

Table 4 presents some representative studies in the sleep posture recognition methods. Since it is a novel method to recognize sleep posture using ECG signal measured by flexible CC electrodes, it is inappropriate to directly compare the classification accuracy between our work and other researches. Pressure-sensing mats with large number of sensors in reference [23, 24, 33] may cause hardware complexity and wire clutter, and data size in reference [34] was too small. As mentioned above, reference [26] is the only similar study we have found. ECG signal was measured using 12 electrodes made of hard materials, which may disturb natural sleep. In reference [35], hard CC electrodes were also used to detect sleeping positions. Unlike reference [26], it was based on changes in capacitance distribution when changing body posture, rather than cECG. In comparison with other works, our work is the only one that can unobtrusively monitor sleep posture with very few sensors. Although the accuracy of our method is not the best of all the studies listed, the gap between them is not large. Moreover, the smart mattress we used can unobtrusively obtain ECG signal throughout the night. The promising results of the overnight experiment showed that the proposed method could be able to monitor sleep posture in real sleep scenarios with considerably high accuracy.

**Table 4:** The comprehensive comparison between our method and other research

References	Sensor type	Number of sensors	Data size	Number of identified postures	ACC (%)	Kappa	Notes
[23]	Pressure sensors	64 × 32	13 subjects	3 (left, right and supine)	82.7	–	Large amount of sensors
[24]	Textile pressure sensors	64 × 27	12 subjects	4 (left, right, supine and prone)	97.9	0.972	Complex system design
[26]	Hard CC electrodes	13	13 subjects	4 (left, right, supine and prone)	98.4	0.967	Hard materials
[33]	Pressure sensors	14 × 32	180 subjects	3 (left, right, and s/p, i.e., supine and prone were merged as one)	94.1	0.866	Large amount of sensors
[34]	Long-narrow force sensors	16	2 subjects	3 (left, right and supine)	78.7	0.681	Data size is too small
[35]	Hard CC electrodes	20 × 15	5 subjects	3 (left, right and supine)	92.76	–	Hard materials
Proposed method	Flexible CC electrodes	3	15 subjects	3 (left, right and supine)	96.2	0.943	Non-contact soft materials

It was worth noting that the classification based on 30-s segment demonstrated superior performance over one heart beat in both short-term experiment and overnight experiment. It suggested that a relatively longer time-length may help the classifier model to extract more features in comparison with a single heartbeat and conclusions drawn from a longer period (such as 30 s) may be of more clinical value.

There are several limitations in this study. First, only three sleep postures were focused on and the prone posture was not studied. It has an important effect on gas exchange, and is of great significance for daily monitoring and early warning of patients with sleep apnea syndrome and cardiovascular diseases. Although prone posture is relatively rare, it should also be taken into consideration in the follow-up work. Second, the data size of our study, especially in the overnight experiment, was relatively small, and the experiments were implemented in the sleep lab. Further validation with larger samples in a variety of environments, including but not limited to sleep labs, student dormitories, and family bedrooms, is warranted. Third, respiratory signals were not extracted from the mattress signals by baseline extraction, heart rate variability calculation, and R-wave amplitude. In future work, joint analysis of respiration signals and ECG signals will be carried out to improve the performance of sleep posture recognition.

## Conclusion

This paper provided a novel method for sleep posture recognition based on capacitive ECG signals. A smart mattress embedded with only three flexible electrodes was introduced first. It can unobtrusively measure two channels of cECG signal through clothes without privacy concerns. Then two sets of experiments (the short-term experiment and the overnight experiment) were designed to evaluate the effectiveness of the proposed system for sleep posture recognition. The overall accuracy of 96.2% was achieved in the short-term experiment was, while the accuracy was 91.0% in the unconstrained overnight experiment. In comparison with existing studies, the proposed system can achieve a considerable classification performance unobtrusively and comfortably. The results suggested that the CC electrode-based smart mattress is potentially promising in sleep posture recognition and sleep quality assessment.

## Methods

### Capacitive ECG and sleeping positions

**Fig. 3:**
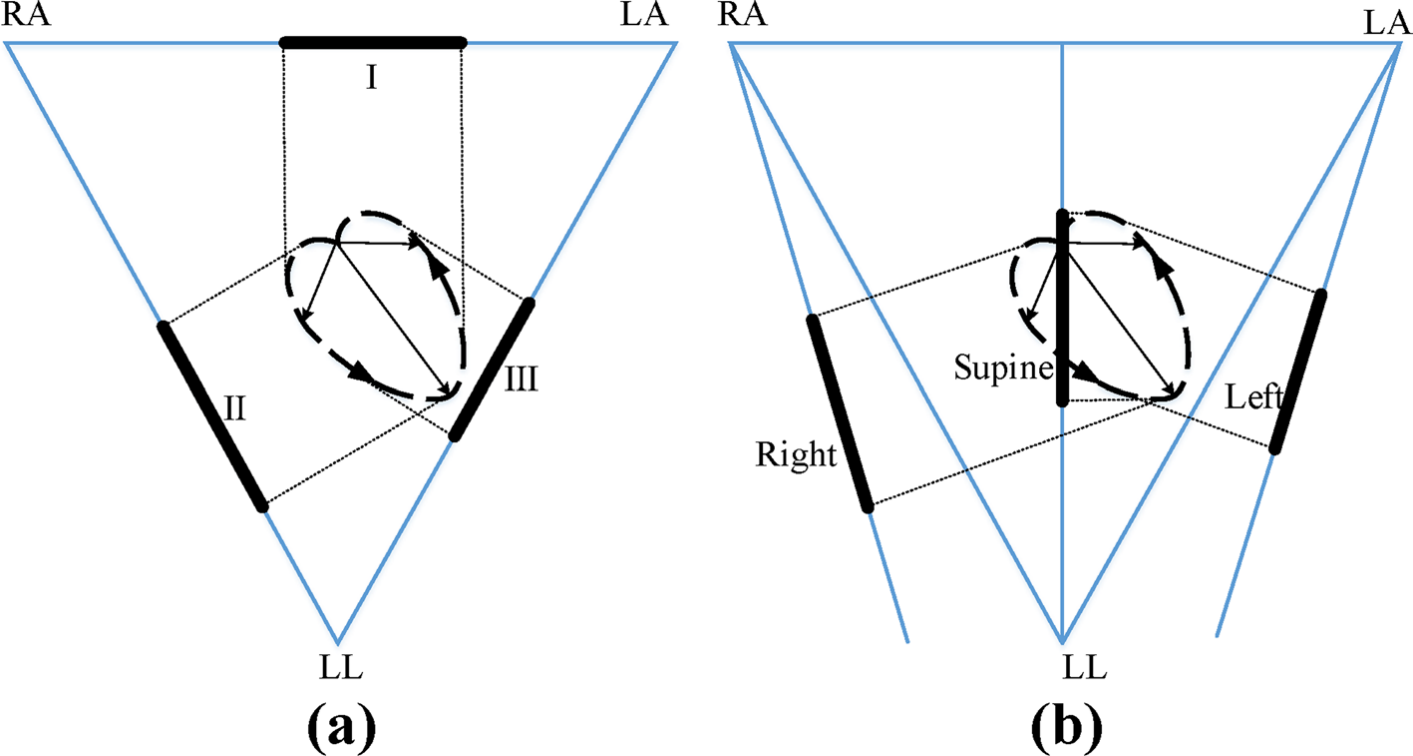
ECG vector projection onto the three limb leads (leads I, II and III) (**a**) and the cECG of three sleep postures (**b**) [36]. The ring-shaped dotted line represents the ECG vector during ventricular depolarization. Three sleep postures include supine, left lateral and right lateral

ECG is the projection of cardiac electrical activity on the body surface. Since the electrodes are attached to different positions on the body surface, ECG waveforms vary in different leads. Figure 3a shows the projections of electric vector during ventricular depolarization in three standard leads. Similarly, according to reference [32], the CC electrodes fixed on the mattress contact different positions of the body through clothes when subjects sleep with different postures. As shown in Fig. 3b, projections of electric vector changes as the sleep postures change. In this work, data mining techniques were used to discover the underlying differences in different ECG vector projections, so as to distinguish cECG signals into three categories, namely supine, left lateral and right lateral.

### System design

**Fig. 4:**
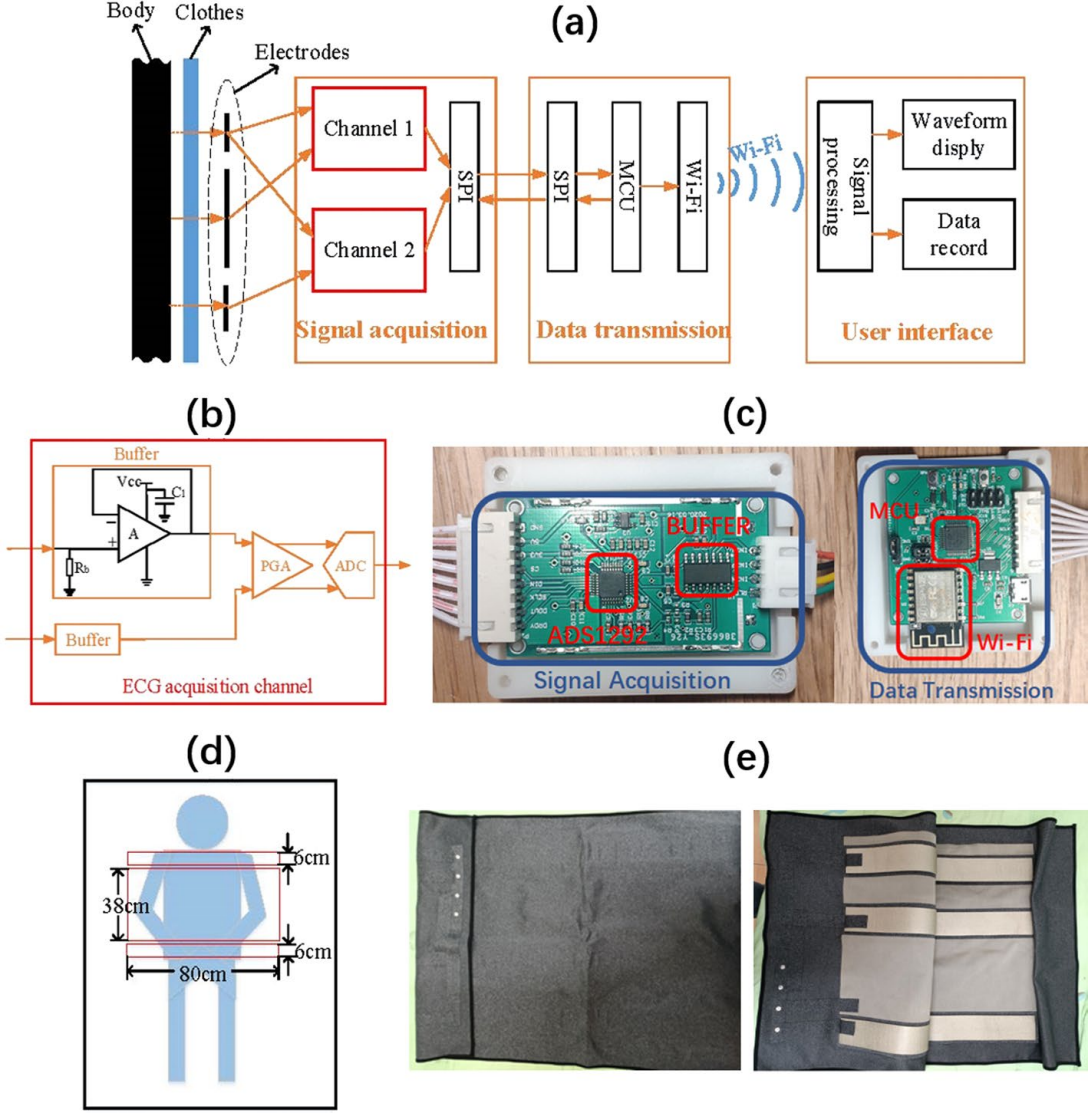
Smart mattress: **a** the system frame; **b** ECG acquisition channel; **c** the hardware of data acquisition and transmission; **d** the mattress structure; **e** the prototype of the smart mattress

A mattress that can monitor ECG signals unobtrusively is proposed. The mattress system consists of four modules, namely CC electrodes, signal acquisition module, data transmission module, and user interface, as shown in Fig. 4a. Three CC electrodes are embedded in the mattress and the signal acquisition module is used to detect and convert the ECG from the analog signal to the digital signal. Then, ECG signals are transmitted from signal acquisition module to microcontroller unit (MCU) through serial peripheral interface (SPI) and finally transmitted to user interface by Wi-Fi. Through the user interface, we can process ECG signals, observe ECG waveforms and record ECG data in real-time.

The signal acquisition module is composed of buffers designed by an operational amplifier (AD8606, Analog Device Inc., Norwood, MA, USA), a programmable gain amplifier (PGA), and an analog-to-digital converter (ADC), as shown in Fig. 4b. The PGA, the ADC, and the SPI are implemented by an integrated circuit ADS1292 (Texas Instruments, Dallas, Texas, USA), which has two analog signal channels and is very suitable for ECG acquisition at low cost. The data transmission module is composed of the MCU and Wi-Fi module. The hardware prototype of the proposed system is shown in Fig. 4c.

The silver fiber conductive fabric is chosen as the main material of the electrodes due to its flexible and user-friendly characteristics. The electrodes length is 80 cm, which ensures the stability of unconstrained ECG measurement during the whole night even if the subject may move or change sleep postures frequently. Besides, the electrodes are flexible and thin (only about 0.1 cm), and will not disturb the user's sleep. The detailed information about size and arrangement of three electrodes is shown in Fig. 4d and the prototype of the smart mattress is shown in Fig. 4e.

### Data collection

We designed two sets of experiments, the short-term experiment and the overnight experiment. Both experiments were conducted in a sleep lab at the Center for Intelligent Medical Electronics, Fudan University. The experiment was a non-clinical study without any harmful procedure and followed the principles of the Declaration of Helsinki strictly. All subjects were required to read experimental instructions and sign informed consent before the experiment.

#### The short-term experiment

**Fig. 5:**
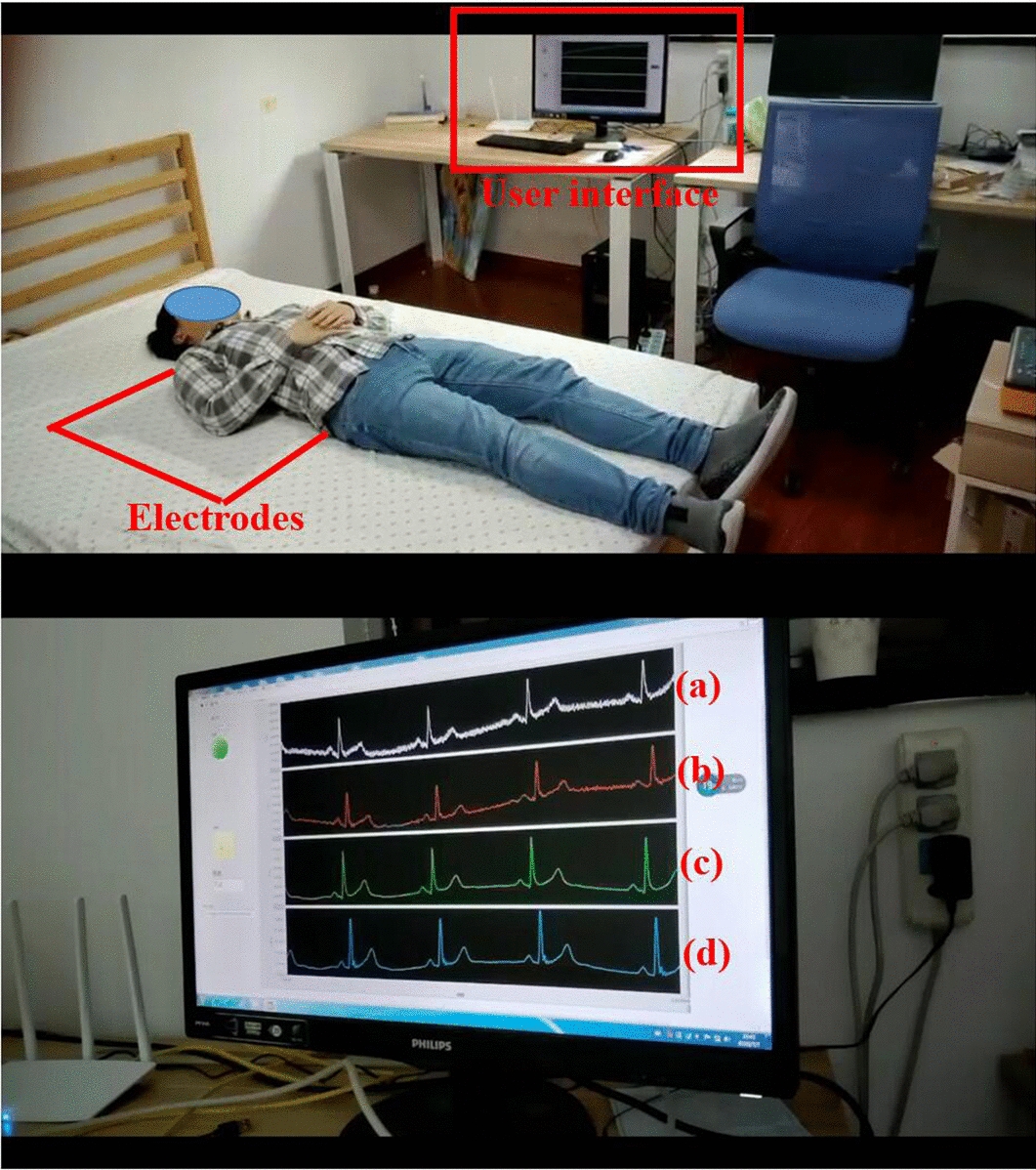
The experimental scene images: **a** raw signal of channel 1; **b** filtered signal of channel 1; **c** raw signal of channel 2; **d** filtered signal of channel 2

Fifteen healthy subjects (10 males, 5 females) aged between 21 and 35 volunteered to participate in the first experiment. The subjects came to the sleep lab wearing their own cottas or shirts. Prior to formal recording, each subject was asked to lie in supine position on the smart mattress for at least 3 min to allow the stabilization. During the experiment, the subjects lay in three sleep postures (including supine, left lateral and right lateral) according to their own habits. After enough relaxation, they kept each sleep posture for 5–6 min and the experiment operator recorded the cECG signals and sleep posture information simultaneously using the user interface. The sampling frequency of cECG signal was set to 500 Hz. The experimental scene is shown in Fig. 5.

#### The overnight experiment

**Fig. 6:**
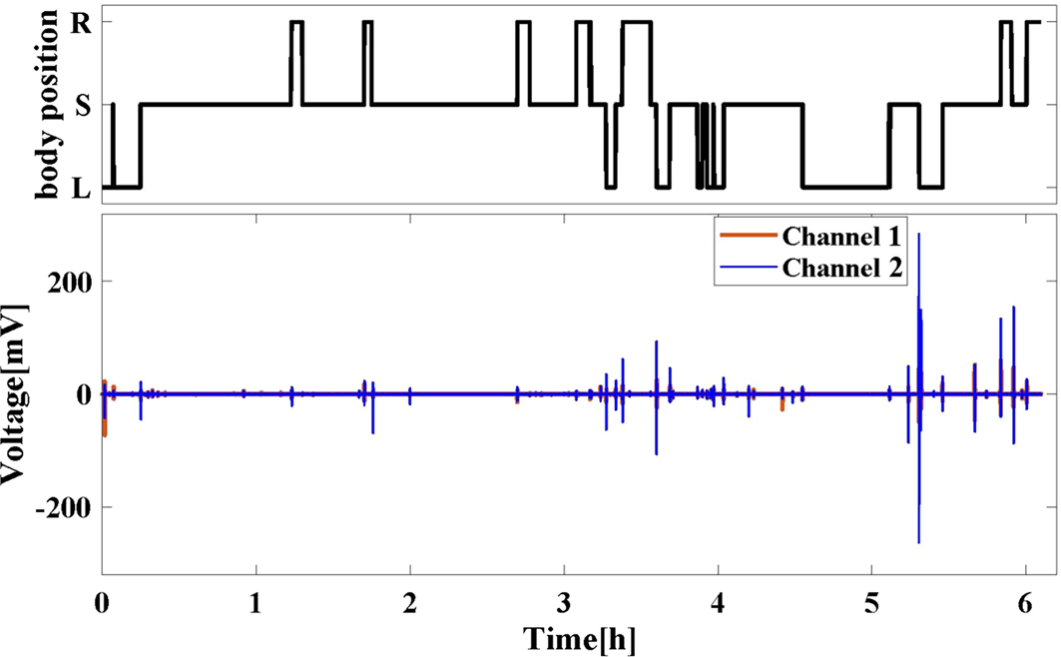
Simultaneously recorded body position signal (L: left lateral. S: supine. R: right lateral) and cECG signal in the overnight experiment

Considering the differences between the data measured under simulated sleep and real sleep, an overnight experiment under unconstrained sleep was carried out to evaluate the performance of the proposed system in a real sleep scenario. A new subject was invited to participate. The testing time was based on the subject’s sleep habits, from about 12:00 pm (midnight) to 6:00 am next day. To record the reference sleep posture, a position sensor of a PSG product (*Grael, Compumedics, Victoria, Australia*) was used. In this experiment, the subject was wearing pajamas and the position sensor of PSG was attached to a chest strap tied to the body. The cECG signals measured by the mattress and sleep postures recorded by PSG overnight are shown in Fig. 6.

### Data preprocessing

For the data of the short-term experiment, first, large motion artifacts caused by changing sleep postures were removed, and 5-min continuous ECG was cut out from the data of each sleep posture, so that the 15-min signal corresponding to the three postures were obtained. In each 5-min ECG signal, all data were retained without specifically removing motion artifacts caused by small movement. Thus, the data set of 15 subjects contains 225 min data, including two channels of ECG signals and labels for 3 sleep postures. Further, raw ECG signals were bandpass filtered with high and low cut-off frequency of 0.5 Hz and 40 Hz, respectively. Then *R*-wave peaks of ECGs were extracted automatically based on multiscale morphological derivative transform [37]. The *RR* interval time series were formed by the intervals of adjacent *R*-wave peaks.

For the overnight experiment, the total test time is about 366 min. All motion artifacts with amplitude exceeding 5 mV were searched and cut out. After removal, the length of effective data for algorithm evaluation is 337 min, accounting for 92.1% of the total test time. Further, the obtained ECG signals were processed according to the above short-term experiment, and were then put into the following classifier.

### Classifier model

In this study, a recurrent neural network (RNN) model based on bidirectional long short-term memory (biLSTM) was used to classify sleep posture. The idea of using RNN model came from the temporal nature of the sleep posture [38] and ECG signals [39]. The long short-term memory (LSTM) network, a special variant of RNNs, has been widely used in time-sequence modeling task. It can capture the temporal dependencies in both short-term and long-term sequences, and avoid the gradient explosion or disappearance commonly existed in artificial neural networks through three control gates in the neuron [40, 41]. The LSTM network can be computed as follows:

4 $${g}_{\mathrm{t}}=\sigma \left({x}_{\mathrm{t}}{W}_{\mathrm{xc}}+{h}_{\mathrm{t}-1}{W}_{\mathrm{hc}}+{b}_{\mathrm{c}}\right),$$

5 $${i}_{\mathrm{t}}={\sigma }_{\mathrm{i}}\left({x}_{\mathrm{t}}{W}_{\mathrm{xi}}+{h}_{\mathrm{t}-1}{W}_{\mathrm{hi}}+{W}_{\mathrm{ci}}\otimes {c}_{\mathrm{t}-1}+{b}_{\mathrm{i}}\right),$$

6 $${f}_{\mathrm{t}}={\sigma }_{\mathrm{f}}\left({x}_{\mathrm{t}}{W}_{\mathrm{xf}}+{h}_{\mathrm{t}-1}{W}_{\mathrm{hf}}+{W}_{\mathrm{cf}}\otimes {c}_{\mathrm{t}-1}+{b}_{\mathrm{f}}\right),$$

7 $${c}_{\mathrm{t}}={f}_{\mathrm{t}}\otimes {c}_{\mathrm{t}-1}+{g}_{\mathrm{t}}\otimes {i}_{\mathrm{t}},$$

8 $${o}_{\mathrm{t}}={\sigma }_{\mathrm{o}}\left({x}_{\mathrm{t}}{W}_{\mathrm{xo}}+{h}_{\mathrm{t}-1}{W}_{\mathrm{ho}}+{W}_{\mathrm{co}}\otimes {c}_{\mathrm{t}-1}+{b}_{\mathrm{o}}\right),$$

9 $${h}_{\mathrm{t}}={o}_{\mathrm{t}}\otimes \sigma \left({c}_{\mathrm{t}}\right),$$

where *x*_*t*_ is the input data and *i*_t_, *f*_t_, *c*_t_ and *o*_t_ represent the input gate, forget gate, cell and output gate, respectively. And *σ* denotes the activation function while *σ*_i_, *σ*_f_ and *σ*_o_ are the logistic sigmoid function of the input gate, forget gate and output gate, respectively. The symbol denotes the scalar product between two vectors. Additionally, *W* and *b* denote the corresponding weight coefficients and bias vectors.

As an improved version of LSTM, biLSTM can read the input from the forward and reverse directions of the data sequence, so as to acquire the contextual semantic information [42]. In our previous work, BiLSTM has been proven an efficient end-to-end approach for noisy photoplethysmography (PPG) segmentation and denoising [43] [44]. This makes biLSTM more suitable for analyzing complex ECG waveforms and extracting significant information from the details.

**Fig. 7:**
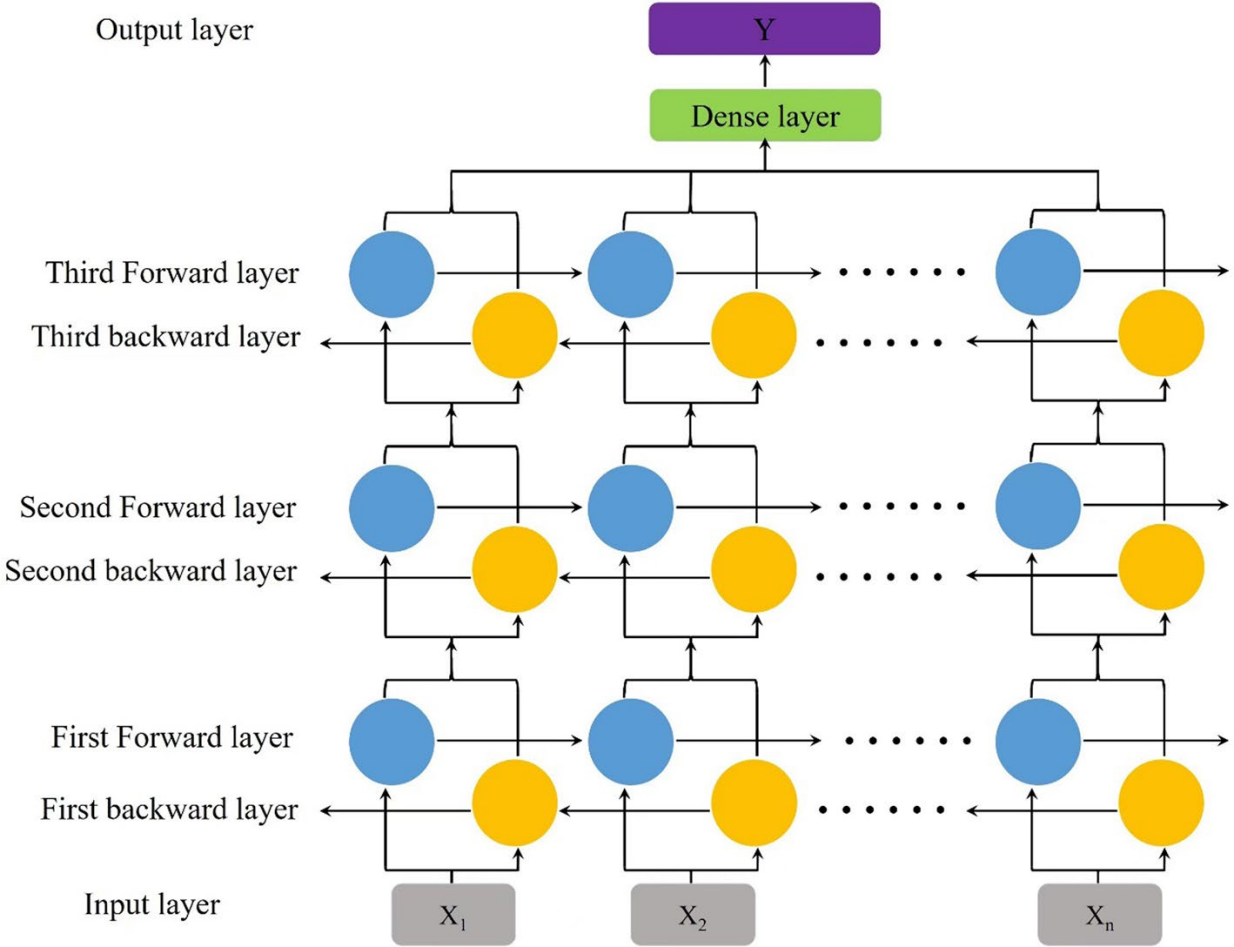
Network structure of the model for sleep posture classification

The model for sleep posture classification contains three biLSTM layers and one dense layer, as shown in Fig. 7. The first biLSTM layer has 200 neurons and two data segments acquired from two channels of cECG signal are input into it. The second and third layers contain 100 neurons and 50 neurons, respectively. The last layer is a dense layer with activation function of SoftMax. It is used to connect the third layer and output the category of the sleep posture, namely supine (${S}_{\mathrm{p}}$), left lateral (${L}_{\mathrm{p}}$) and right lateral (${R}_{\mathrm{p}}$). Detailed training parameters are summarized in Table 5.

**Table 5:** The detailed parameters of the model

Parameter	Value
Input	[ECG_channel1, ECG_channel2]
Output	Category of the sleep posture (*Sp, Lp, Rp*)
Layer number of biLSTM	3
biLSTM size	[200, 100, 50]
biLSTM state activation function	tanh
biLSTM gate activation function	Sigmoid
Output layer	Softmax
Loss function	Cross-entropy loss function
Optimizer	Adam
Number of training epochs	150

In previous sleep posture studies based on ECG signal, the input signal segment was RR interval or segment with different window lengths. The window length of the input signal segment in reference [26], the only study we found on sleep posture recognition based on CC electrodes, is 30 s. Considering that 30-s length is the epoch length usually used for sleep staging, we defined two types of window length of the input signal, namely one heart beat (i.e., RR interval) and 30 s in this study. Each cECG segment was normalized and resampled to 250 samples to avoid different lengths of different input segments due to variable heart rate. Taking the dataset of the short-term experiment as an example, the 5-min data of each subject in one sleep posture were divided to 10 data segments with the length of 30 s, and only one sleep posture prediction was estimated in each 30-s segment. If the outputs of the proposed classifier in each 30-s segment contain two or more sleep postures, the posture with the most occurrences is chosen as the final prediction.

## Data Availability

The datasets used and analyzed in the current study are available from the corresponding author on reasonable request.

## Abbreviations

CC: Capacitively coupled electrode
cECG: Capacitive ECG
RNN: Recurrent neural network
HR: Heart rate
HRV: Heart rate variability
PSG: Polysomnography
MCU: Microcontroller unit
SPI: Serial peripheral interface
PGA: Programmable gain amplifier
ADC: Analog-to-digital converter
LSTM: Long short-term memory
biLSTM: Bidirectional LSTM
LOSOCV: Leave-one-subject-out cross-validation
TP: True positive
FP: False positive
TN: True negative
FN: False negative
